# HIV Treatment as Prevention: The Utility and Limitations of Ecological Observation

**DOI:** 10.1371/journal.pmed.1001260

**Published:** 2012-07-10

**Authors:** M. Kumi Smith, Kimberly A. Powers, Kathryn E. Muessig, William C. Miller, Myron S. Cohen

**Affiliations:** 1 Department of Epidemiology, University of North Carolina, Chapel Hill, North Carolina, United States of America; 2 Department of Medicine, University of North Carolina, Chapel Hill, North Carolina, United States of America; 3 Department of Microbiology and Immunology, University of North Carolina, Chapel Hill, North Carolina, United States of America; Duke University Medical Center, United States of America

## Abstract

Results from several observational studies of HIV-discordant couples and a randomized controlled trial (HIV Prevention Trials Network 052) show that antiretroviral therapy (ART) can greatly reduce heterosexual HIV transmission in stable HIV-discordant couples. However, such data do not prove that ART will reduce HIV incidence at the population level. Observational investigations using ecological measures have been used to support the implementation of HIV treatment for the specific purpose of preventing transmission at the population level. Many of these studies note ecological associations between measures of increased ART uptake and decreased HIV transmission. Given the urgency of implementing HIV prevention measures, ecological studies must de facto be used to inform current strategies. However, the hypothesis that widespread ART can eliminate HIV infection may have raised expectations beyond what we may be able to achieve. Here we review and discuss the construct of the exposure and outcome measures and analysis methods used in ecological studies. By examining the strengths and weaknesses of ecological analyses, we aim to aid understanding of the findings from these studies to inform future policy decisions regarding the use of ART for HIV prevention.

## Introduction

Ecological studies use observational data to examine relationships between exposures and outcomes at the level of groups rather than individuals [1]. When individual-level data are unavailable, ecological studies can provide important insight into population-level trends [2],[3]. Ecological studies appeal to researchers and policy-makers because they are inexpensive, use existing data, and are applicable to a broad range of issues. However, statistical models using only group-level data cannot evaluate person-level details and are therefore unable to test etiological hypotheses [2],[4]–[6]. Further, because ecological studies often use separate data sources to measure exposures and their potential effects, the link between exposures and outcomes cannot be determined at the individual level.

Concern over these limitations has focused on “ecological fallacy,” in which associations detected at the population level are mistakenly interpreted to reflect the experience of individuals in that population [1]. The first study describing ecological fallacy presented an analysis of literacy and immigration in the US, in which states with higher proportions of immigrants were shown to have higher average literacy rates [7]. An “ecologically fallacious” interpretation of this association would be that immigrants have higher literacy rates than native-born individuals; in fact, individual-level analysis shows lower literacy rates among immigrants. The best explanation for this particular population-level observation is that immigrants tend to settle in sites where the native-born individuals have higher literacy levels [7].

Despite their limitations, ecological studies play an important role in generating hypotheses that can be tested in experimental or individual-level observational studies [2],[8]. For instance, ecological analyses were successfully applied during the exploratory phases of research on male circumcision to prevent HIV, in which geographical associations between circumcision rates and HIV prevalence [9]–[11] provided the foundation for two decades of observational research [12],[13] on the topic. All three randomized clinical trials that followed were halted because of a readily demonstrable reduction of HIV acquisition in circumcised men [14],[15]. A Cochrane review published in 2009 concluded that male circumcision is a clinically viable HIV prevention strategy [16].

Here we describe an illustrative set of observational studies that use ecological measures to examine the population-level effects of antiretroviral therapy (ART) on HIV transmission. We critically review what these studies measure, how they measure it, and how their findings are interpreted. These results are used to provide insight into the strengths and limitations of this approach.

## Ecological Studies of ART for HIV Prevention

The narrative of exploring the effects of treatment on prevention shares similarities with the narrative for male circumcision, though with a somewhat different chronology. The hypothesis that antiretroviral agents can prevent sexual HIV transmission was suggested in 1988 shortly after the development of azidothymidine, which was found to effectively penetrate the genital tract [17]. This report was followed by more intensive study of the effect of newly developed antiretroviral agents on HIV replication in the male and female genital tract [18]–[21]. In 1994, Musicco et al. observed that azidothymidine could reduce transmission of HIV in a cohort of discordant couples by 50% [22]. Several clinical trials in the late 1990s showing that ART stopped mother-to-child transmission lent further credibility to the potential use of ART to prevent sexual transmission [23]. In 2000, a randomized clinical trial, HIV Prevention Trials Network 052 (HPTN 052), was launched to determine the magnitude and durability of the effect of combination antiretroviral agents on the prevention of sexual transmission of HIV [24]. After this trial was launched, several [25]–[28], but not all [29], individual-level observational studies reported a protective effect of ART against HIV transmission in serodiscordant couples. In addition, many modeling exercises suggested varying degrees of population-level prevention benefit from broader use of ART [30], the most widely discussed of which predicted elimination of HIV within five years under ideal conditions [31]. These models are discussed in a review by Eaton et al. [32] in the July 2012 *PLoS Medicine* Collection, “Investigating the Impact of Treatment on New HIV Infections.” A third group of eight ecological studies examined the population-level effects of widespread ART on HIV incidence using ecological measures, and reported significant effects [33]–[38] in all but two cases [39],[40]. These eight studies are the focus of this review. Finally, in mid-2011, the HPTN 052 investigators reported a 96% reduction of HIV transmission in heterosexual couples over the 1.7 years of follow-up [41].

The promise of ART to control—and perhaps even eliminate [31]—HIV has mobilized calls from public health leaders to integrate preventive and clinical applications of ART [42]–[45]. In light of several trials showing markedly improved survival for those initiating ART earlier in the course of disease, such initiatives often emphasize the clinical benefits that early treatment can bring HIV-infected persons [46],[47]. However, numerous behavioral, epidemiological, and programmatic challenges may well limit the ability to translate the individual-level prevention benefits of ART to a larger population [48]–[52]. As such, demonstration of a minimally biased population-level benefit is critical. Not surprisingly, there is a credible tension between the need for more randomized individual- and community-level trials (also called cluster randomized controlled trials), and the immediate scale-up of HIV treatment to prevent the spread of HIV [53]–[55]. The arguments for immediate and broader roll-out of ART for the sake of prevention are based on the HPTN 052 study [41], observational studies of transmission within HIV-discordant couples [25]–[29], ecological reports [33]–, and modeling exercises [31],[56]–[59].

In this report we examine eight influential ecological studies that assess the population-level effects of ART on HIV transmission (Table 1). Most of the studies are from North America [33],[34],[36],[38]–[40], with one set in Taiwan [35] and one in Australia [37]. Each study uses an ecological measure of the exposure, such as access to ART, or the outcome, such as HIV incidence, or both (summarized in Table 1; further considerations detailed in Table 2).

**Table 1:** Summary of exposure and outcome measures in studies using ecological measures to assess population-level effects of ART on HIV transmission.

Study (Year)	Study Location	Exposure: Trends in Population-Level Infectiousness			Outcome: Trends in HIV Transmission		
		Assessment	Measure	Trend Direction	Assessment	Measure	Trend Direction
Castel et al. [39] (2011)	Washington, D. C., US	Population in clinical care	Annual mean and total CVL; portion with undetectable VL	↓; ↑	Sentinel surveillance	Annual numbers of newly diagnosed cases	→
Das et al. [34] (2010)	San Francisco, US	Population in clinical care	Annual mean and total CVL	↓	Sentinel surveillance	Annual numbers of newly reported HIV diagnoses; annual incidence rate estimated from surveillance data using STARHS (trend was not statistically significant)	↓; ↓
Fang et al. [35] (2004)	Taiwan (national)	Time period	Time period (pre- versus post-ART period)	—	Sentinel surveillance	Surveillance data used to calculate average annual HIV transmission rate (new cases/prevalent cases)	↓
Katz et al. [40] (2002)	San Francisco, US	Population in clinical care	Annual prevalence of ART use in HIV-infected MSM identified in HIV/AIDS registry	↑	Convenience sample of MSM	STARHS-estimated incidence using data from a VCT clinic and a STD clinic	→; ↑
Law et al. [37] (2011)	Australia (national)	Population in clinical care	Annual portion of treated patients with undetectable VL	↑	No direct assessment	Reference to previous publication describing HIV incidence trends during the same study period	↓
Montaner et al. [33] (2010)	British Columbia, Canada	Population in clinical care	Annual numbers of HIV patients receiving HAART; annual mean CVL	↑; ↓	Sentinel surveillance	New HIV-positive tests per 100 population	↓
Porco et al. [38] (2004)	San Francisco, US	Probability sample of MSM	Predicted per contact infectivity during the pre- and post-ART periods	↓	Probability sample of MSM	Annual testing in a cohort of HIV-negative MSM	↓
Wood et al. [36] (2009)	Vancouver, Canada	Convenience sample of IDUs	Biannual median CVL	↓	Convenience sample of IDUs	Annual testing in a cohort of HIV-negative IDUs	↓

↑, upward trend; ↓, downward trend; →, stable rate. For studies using two exposure or outcome measures, two arrows are shown, corresponding to the measures listed first and second.

HAART, highly active ART; STD, sexually transmitted disease; VCT, voluntary counseling and testing; VL, viral load.

**Table 2:** Summary of measures used and considerations for their use.

Measure	Considerations
**Exposure: trends in population-level exposure to suppressive ART**	
Before/after ART	Dichotomous measure does not quantify the level of suppressive ART use in a population
Prevalence of ART use	Only represents the prevalence of ART use among populations in clinical care; does not account for failure to achieve viral suppression
Portion of treated individuals with undetectable VL	Only represents the portion of individuals with undetectable VL among populations in clinical care
CVL	Only represents VL among populations in clinical care; aggregate and mean values obscure important differences in transmissibility among individuals
**Outcome: trends in HIV transmission**	
New HIV diagnoses	New HIV diagnoses do not necessarily represent incident cases.
HIV incidence via longitudinal cohort follow-up	Individuals who enroll and stay in cohorts may have lower HIV incidence than those who do not; choice of testing interval and assay can introduce bias
HIV incidence via laboratory-based methods for identifying recent infections	Recent infections identified only among HIV-infected individuals who test; assays have low specificity and can overestimate recent infections
HIV transmission rates (new cases per prevalent case-year) from modified back-calculation approach	Sensitive to peculiarities of a population's testing behavior, including frequent repeat testers or variable rates of disease progression among identified cases

VL, viral load.

## Measuring Population Exposure to ART

The simplest way investigators have characterized ART exposure in a population of HIV-infected persons is to use a dichotomous “before/after” measure, as in the case of Fang et al. [35] and Porco et al. [38], based on the time at which scale-up of local HIV treatment policies improved access to ART. Other investigators have used more detailed measures of ART exposure, including Montaner et al. [33], who estimated the number of HIV-infected persons known to be receiving ART in a population, or Katz et al. [40], who used prevalence of ART use among all identified HIV patients. How well these measures reflect actual ART exposure of an entire HIV-infected population depends on the extent to which some subpopulations remain “hidden” to investigators. ART exposure of the entire HIV-infected population can only be measured if every person with HIV infection can be identified and their treatment status assessed.

**Figure 1:**
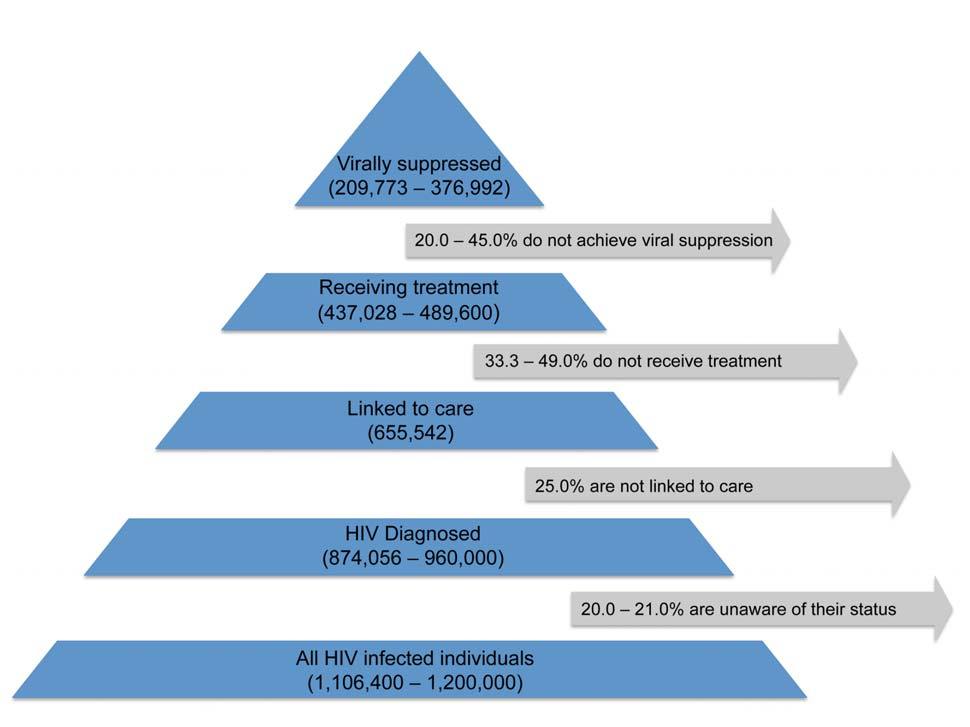
Estimated numbers of HIV-infected individuals in the US retained (and corresponding percentages lost) at various stages of the test, link, and treat cascade. This figure is based on data from [61],[62].

The hypothesis that population ART usage will decrease HIV incidence relies on the assumption that ongoing HIV care will sustain viral suppression, which is essential to transmission prevention [60]. However, large numbers of HIV-infected persons are lost to follow-up along the path from testing to suppressive treatment [61],[62]; the US Centers for Disease Control and Prevention recently estimated that only about 24% of the 1.2 million people in the US with HIV infection in 2010 were virally suppressed (Figure 1) [61]–[63]. Even once in care, rates of treatment refusal by eligible individuals can be substantial [64].

To address the shortcomings of measures that do not fully reflect *suppressive* ART use, alternative metrics incorporate viral load information, based on the well-understood relationship between sexual transmission of HIV from infected individuals and viral concentrations in their blood [65] and genital fluids [66]. One such measure uses the proportion (or absolute number) of treated individuals in a study population with undetectable viral load, usually defined as having fewer than 400 copies/ml [33],[37]–[39].

A related measure, community viral load (CVL), is used by Das et al. [34], Montaner et al. [33], and Wood et al. [36], and is defined as the total, mean [33],[34], or median [36] viral load for a particular group or geographic region in a given period of time (usually a year). CVL may be a useful biomarker for describing population-level treatment outcomes over time, particularly in cases in which geospatial information about the patients' primary residence or point of medical care is available, allowing investigators to compare geographic disparities in CVL with other predictive factors such as socioeconomic status or proximity to health care programs [34],[39]. However, because most CVL measures rely on public health surveillance data [33],[34],[39], these exposure measures reflect the treatment outcomes only for the subset of the HIV-infected population who get tested for HIV, link to care, and remain in care long enough to contribute such measurements. Patients with acute infection unidentified by serological testing are de facto not considered in the calculation of CVL, but may well be expected to contribute disproportionately to onward HIV transmission [67],[68]. Additionally, the use of an aggregate measure of viral load in a community cannot capture other important drivers of HIV transmission, such as the distribution of viral loads within the population, sexual and drug-using behaviors, and the sexual or drug-use networks through which these behaviors spread HIV.

## Outcome Assessment: Tracking Population HIV Transmission

Accurate assessment of HIV incidence is critical for evaluating the population-level effect of interventions, but such assessment is challenging. The simplest approach is taken by Law et al. [37], who simply refer to HIV incidence trends cited in past publications [37]. Another approach, taken by Montaner et al. [33], Das et al. [34], and Castel et al. [39], estimates population-based incidence from information on newly identified cases, using new HIV diagnoses as a direct proxy for new infections. Obviously, newly diagnosed patients acquired HIV at some unknown earlier time, and so they are not “incident” in the traditional use of the word. Using new diagnoses as a proxy for incidence also misses populations that do not seek testing and that may have lower access to health care and a corresponding higher risk for acquiring HIV [69],[70]. Changes in the number of new HIV diagnoses may reflect actual changes in incidence, but will also be affected by changes in availability of services and testing behaviors [71].

A second population-based incidence estimation method, used by Fang et al. [35], back-calculates past incidence from new diagnoses [35]. This method relies in part on assumptions of uniform parameters for disease progression markers such as the onset of AIDS symptoms or the proportion of newly diagnosed individuals with CD4 cell count less than 200 cells/µl, although variation in the rate of decline of CD4 cell count over time and across gender, ethnicity, and HIV subtypes undermines the validity of this method [72]–[74].

Laboratory assays to identify persons with recent HIV infection can be applied to stored biospecimens collected in the course of routine surveillance or epidemiological research studies and may provide a more rigorous method to determine current HIV incidence from new diagnoses. The serologic testing algorithm for recent HIV seroconversion (STARHS) [75] derives current HIV incidence from the prevalence of recent infections, based on the assay window period, delineated by the seroconversion dates as detected by the original HIV-1 antibody test and the STARHS method, and adjusting for the estimated prevalence among non-testers and the probability that HIV-infected individuals will test, receive treatment, and/or have missing specimens. Although the investigations that use this method take advantage of existing surveillance data, as in the case of Das et al. [34] and Katz et al. [40], logistical challenges in storing and tracking remnant blood can affect the completeness of data. Furthermore, even relatively new laboratory methods such as the detuned enzyme-linked immunosorbent assay or the newer BED capture enzyme immunoassay have been known to misclassify established infections as incident infections; therefore, results must be interpreted with some caution [76]. The use of this approach has generally fallen out of favor pending development of better laboratory-based tests or algorithms [77].

In contrast to population-based methods, longitudinal cohort follow-up data have also been used to define population incidence, as in the analyses carried out by Wood et al. [36] and Porco et al. [38]. Although long considered the gold standard of HIV incidence estimation, cohort follow-up is not immune to bias, such as that which can result from the choice of testing intervals and HIV assay [78]. Moreover, cohort participants may be a poor proxy for the rest of the population, especially if the individuals who enroll and remain in the cohort have fewer risk behaviors than their unobserved counterparts.

## Identifying the Effects of ART on HIV Transmission

The ultimate aim of these investigations is to determine whether population-level ART exposure has affected HIV transmission. Some investigators used inductive reasoning to synthesize either their own results [38],[40] or a combination of their own results and other published reports [37]. The remaining studies quantify the association by comparing transmission rates, defined as the ratio of new cases to prevalent cases in an interval of time, before and after introduction of ART [35], or by using time series regression modeling [33],[34],[36],[39]. Although these methods of analysis differ considerably, it is worth consideration that nearly every study arrives at the same conclusion: that increased population exposure to ART leads to lower HIV transmission (Table 3).

**Table 3:** Analysis methods and conclusions regarding effects.

Author (Year)	Analysis Method	Statistical Analysis Results	Control for Confounders?	Conclusions
Castel et al. [39] (2011)	Negative binomial regression of new diagnoses on mean CVL	No effect estimate given, but lack of association reported (*p* = 0.11)	No	No association was found between trends in the mean CVL and newly diagnosed HIV/AIDS cases
Das et al. [34] (2010)	Poisson regression of new diagnoses on changes in total and mean CVL; meta-regression of estimated incidence on changes in total and mean CVL	No effect estimate given, but statistically significant trend with new diagnoses noted (*p* = 0.003)	Notes reported trends in rectal gonorrhea, but no formal assessment	Reductions in CVL were associated with a decrease in new HIV diagnoses, but not with slight HIV incidence decrease
		No effect estimate given, but lack of association with estimated incidence noted (*p*>0.30)		
Fang et al. [35] (2004)	Modified back-calculation to estimate reduction in transmission rate (new cases per prevalent case-year) between pre- and post-HAART eras	Pre-HAART transmission rate estimated as 0.391 new infections per prevalent case	Secondary analysis of concurrent trends in annual reported cases of syphilis and gonorrhea, but no formal assessment	Provision of free ART was associated with a 53% reduction in the estimated HIV transmission rate
		Post-HAART transmission rate estimated as 0.184 new infections per prevalent case		
Katz et al. [40] (2002)	Inferences drawn from observation of concurrent changes in HIV incidence rates, reported sexual behavior, STI diagnoses, and ART use among population in clinical care	—	Secondary analysis of concurrent trends in reported risk behaviors and cases of rectal gonorrhea among MSM, but no formal assessment	ART impact on HIV transmission has been counterbalanced by increased reported risk behaviors
Law et al. [37] (2011)	Inferences drawn from predicted changes in prevalence of undetectable VL among population in clinical care and external reports of HIV incidence	—	No	Declines in predicted detectable VL between 1997 and 2009 coincide with reports of rising new diagnoses and estimated incidence in the same community
Montaner et al. [33] (2010)	Poisson regression of estimated new diagnoses on changes in median CVL and numbers receiving HAART	Effect of 100 new patients receiving HAART on estimated new diagnoses predicted as −0.97 (95% CI 0.96–0.98)	Notes reported trends in infectious syphilis, rectal gonorrhea, and genital chlamydia as proxies for sexual risk behaviors; trends in hepatitis C were also noted as proxy for unsafe injecting behaviors	Increased ART coverage and reduced CVL are associated with a decreased number of new HIV diagnoses
		Effect of 1 log decrease median CVL on estimated new diagnoses predicted as −0.86 (0.75–0.98)		
Porco et al. [38] (2004)	Inferences drawn from trends in annual HIV incidence based on antibody testing and time period (pre- versus post-HAART period) as indicator of ART use	—	Transmission probability accounts for sexual risk behaviors among surveyed MSM	Wider availability of ART appears to have slowed transmission in the study population
Wood et al. [36] (2009)	Unadjusted and adjusted Cox proportional hazards regression of time to seroconversion on median CVL in the preceding six months	Unadjusted hazard ratio for effect of median CVL on time to seroconversion estimated as 3.57 (2.03–6.27) per log_10_ CVL increase	Adjusted model controlled for needle sharing, unprotected sex, ethnicity, daily heroin use, and unstable housing	Median CVL predicts HIV incidence independent of HIV risk behaviors
		Adjusted hazard ratio for effect of median CVL on time to seroconversion estimated as 3.32 (1.82–6.08) per log_10_ CVL increase		

CI, confidence interval; HAART, highly active ART; STI, sexually transmitted infection; VL, viral load.

However, inaccurate assessment of exposures or outcomes can generate bias [51]. Overestimating the decline in incidence, for instance—perhaps because of an unrecognized change in testing behaviors—could produce an upward bias in the estimated impact of ART on HIV transmission. Additionally, statistical associations do not show causation, and observed trends in HIV diagnoses may be due to factors other than population-level exposure to ART. For example, declines in HIV incidence in settings worldwide—most of which started to occur before ART was available or could be expected to have had an effect—have been ascribed to various phenomena, including the saturation of HIV in high-risk groups [79] and changes in sexual behavior in response to the HIV pandemic [71],[78]. Although the potential confounding effects of changes in HIV-related risk behaviors have been widely acknowledged, only one report, from Vancouver [36], formally controls for them in a regression model (Table 3). By comparison, another study from British Columbia attributes large numbers of averted HIV infections among injection drug users (IDUs) to broader uptake of ART in the community, but some have suggested that the analysis underestimates the potential protective effects of other HIV prevention measures directed at the same community [80]. Indeed, the protective effects of Vancouver's safer injection sites have been documented in the past [81],[82]. Also, consistent ART adherence may be difficult to sustain in IDUs [83], further suggesting that factors beyond viral suppression may have contributed to the reduction in HIV incidence in this population.

The ability of ART to visibly reduce the number of newly diagnosed cases of HIV takes time, because most new diagnoses are made years after infection occurs, and many patients present with a reduced CD4 count, reflecting substantial progression of HIV disease. But in some ecological studies, the effect of ART is presumed to be almost immediate. In the report from British Columbia [33], where combination ART was introduced in 1996, the largest decrease in documented new HIV diagnoses took place between 1997 and 2000, but it is reasonable to question whether enough suppressive combination ART was immediately available to most patients to explain this decline.

## Alternative Results and Other Considerations

The comparative lack of reports investigating the ecological effects of population-level ART in settings where rising incidence rates have been detected [84],[85] suggests potential publication bias. It is also noteworthy that ecological studies of ART for HIV prevention are almost exclusively from developed western settings, likely because of the limited availability of surveillance data, viral load measurements, or registry data in resource-constrained settings.

Stable or rising HIV incidence among certain population subgroups with ready access to ART suggests the possibility that identified relationships between ART access and declines in HIV diagnoses in the studies reviewed here may be overstated. For example, HIV incidence (estimated by STARHS) increased and then stabilized among voluntary testers in San Francisco between 1999 and 2006 [85], and model-estimated numbers of new HIV infections among men who have sex with men (MSM) in British Columbia increased by 13% from 2005 to 2008 [86]. In parts of Australia, the number of HIV diagnoses among MSM between 2000 and 2006 doubled, although cohort data suggest that this observation may be largely driven by new infections among older MSM [87],[88]. In Denmark [89] and the UK [90], incidence rates among MSM have reportedly increased. In Canada, some subgroups of IDUs have experienced rising HIV incidence, including Aboriginals [91], women [92], and youth [93], prompting a call for renewed prevention efforts [94].

More than 50 experimental studies of treatment as prevention are in some stage of development, and more can be anticipated [95],[96]. Policy-makers often do not have the luxury of waiting years for trial data, and all decisions take place under a certain degree of uncertainty. To this end, several studies, including some considered in this review, have successfully applied novel tools of geospatial mapping and phylogenetic analysis to aid interpretation of observational data. A study in the UK [97] used viral molecular phylogeny to determine the single most likely transmitter among MSM, allowing the investigators to account for the higher transmission probability of individuals with acute and early infection. Other studies, including those from San Francisco [34] and Washington, D. C. [39], used geospatial analysis to illustrate the spatial distribution of HIV-infected individuals in communities. Most recently, investigators at the Africa Centre for Health and Population Studies in South Africa have been able to identify a relationship between the density of ART use and HIV acquisition risk within a community by studying HIV incidence in a longitudinal cohort of more than 16,000 individuals (personal communication, F. Tanser). Additional strengths of this study include the use of information about the patients' primary residence and attempts to control for at least some possible confounders of the relationship between ART uptake and HIV incidence in the same community.

Several large cluster randomized controlled trials are being developed [98]. A team from the Harvard School of Public Health AIDS Initiative, working with partners in Botswana, will target individuals with sustained, high plasma viral loads for immediate treatment, a strategy that could have exponential public health benefits [73]. A second group from the London School of Hygiene & Tropical Medicine and Imperial College London plans to test the feasibility and impact of a universal test-and-treat strategy along with other combination prevention measures, including male circumcision [99]. But clinical trials have their own limitations, including time, cost, ethical challenges, and perturbations to the underlying community that can cause bias. And there is never a guarantee that approaches employed in a trial will prove effective outside of the trial setting.

## Conclusions

Suppressive ART prevents HIV transmission in stable, monogamous, heterosexual couples. While ART seems to hold great promise as a public health tool, its population-level benefits have not been proven. Although ecological studies can play an important role in the development of new HIV prevention strategies, they are methodologically limited to building justification of further formal scientific inquiry into population-level effects of the potential policies in question. They are therefore the first of many steps in the path from science to policy, beginning with the establishment of biological plausibility, and progressing to assessment of an individual-level effect and then a group-level effect. Though most policy decisions must be made under conditions of uncertainty, the hypothesis that widespread ART can eliminate HIV infection [31],[100] may have raised expectations beyond what can actually be achieved. Additionally, implementation of treatment as prevention is not without its risks, including the rise of population-level drug resistance with the rapid uptake of ART in the face of continued limited infrastructure, and increased risk compensation by treated individuals who believe that treatment alone may justify forgoing other forms of protection [101]–[103].

Although we expect an impact of ART at the population level, the magnitude of the effect may not be as great as some hope; measuring the impact of ART roll-out on HIV spread, as in several planned cluster randomized controlled trials, therefore remains a critical step. Much as combination prevention methods are believed to be better than single interventions for HIV prevention [104], all the methods available to determine the benefits of prevention interventions, including ecological studies, should be deployed. The results must be weighed and used with a full understanding of the methods used to define the outcomes of treatment of HIV infection for prevention of transmission.

**Key Points**

- Several strong observational studies and one randomized controlled trial, HPTN 052, demonstrate that ART reduces the transmission of HIV in stable, heterosexual HIV-discordant couples.
- A number of ecological studies, which use observational data to examine relationships between exposures and outcomes at the level of groups instead of individuals, have found associations between the broader use of antiretroviral agents and reductions in new HIV diagnoses in at-risk populations or in the general population. Ecological studies may generate hypotheses that can be explored using other experimental or observational methods.
- A better understanding of the strengths and limitations of the various exposure and outcome measures used in ecological studies that examine the population-level effects of ART on HIV transmission, and the methods used to analyze them, is essential for the effective application of the findings in policy-making processes.
- Methodological challenges such as measurement error, selection bias, confounding, and assumptions about the time lag of effects must be taken into account when interpreting the results of these studies.
- Prospective measurement of the population-level impact of ART can be approached through cluster randomized controlled trials, but the cost, time, and degree of difficulty of designing and conducting these studies are appreciable, and the potential for the intervention itself to introduce bias can threaten the validity of the results.
- Measuring the population-level benefits of ART is critical to HIV prevention efforts, and consideration of results of all methods may be used to inform ongoing research and public health policy. A firm understanding of the strengths and weaknesses of each approach is crucial to the interpretation of results and allocation of resources.

## Abbreviations

ART: antiretroviral therapy
CVL: community viral load
HPTN 052: HIV Prevention Trials Network 052
IDU: injection drug user
MSM: men who have sex with men
STARHS: serologic testing algorithm for recent HIV seroconversion
